# Simultaneous MQMAS NMR Experiments for Two Half-Integer Quadrupolar Nuclei

**DOI:** 10.1016/j.jmr.2020.106831

**Published:** 2020-11

**Authors:** Samuel J. Page, Angelo Gallo, Steven P. Brown, Józef R. Lewandowski, John V. Hanna, W. Trent Franks

**Affiliations:** a Department of Physics, University of Warwick, Coventry CV4 7AL, United Kingdom; b Department of Chemistry, University of Warwick, Coventry CV4 7AL, United Kingdom

**Keywords:** MQMAS, Solid State NMR, Multiple Receiver, Quadrupolar, Interleaved

## Abstract

A procedure to acquire two Multiple-Quantum Magic Angle Spinning (MQMAS) NMR experiments with the same instrument time is presented. A triply tuned probe is utilized with multiple receivers to collect data with staggered acquisitions and thus more efficiently use the instrument time. The data for one nucleus is collected during the recovery delay of the other nucleus, and vice versa. The instrument time is reduced to 60–80% of the time needed for the single acquisition collection Specifically our approach is presented for recording triple-quantum (3Q) ^17^O and either 3Q or quintuple-quantum (5Q) ^27^Al MAS NMR spectra of a 1.18Na_2_O•5SiO_2_•Al_2_O_3_ glass gel.

## Highlights

• Multiple MQMAS experiments acquired simultaneously.
• Time savings by interleaved experiments.
• Solid State NMR using multiple receivers for materials.

## Graphical abstract

**Figure:**
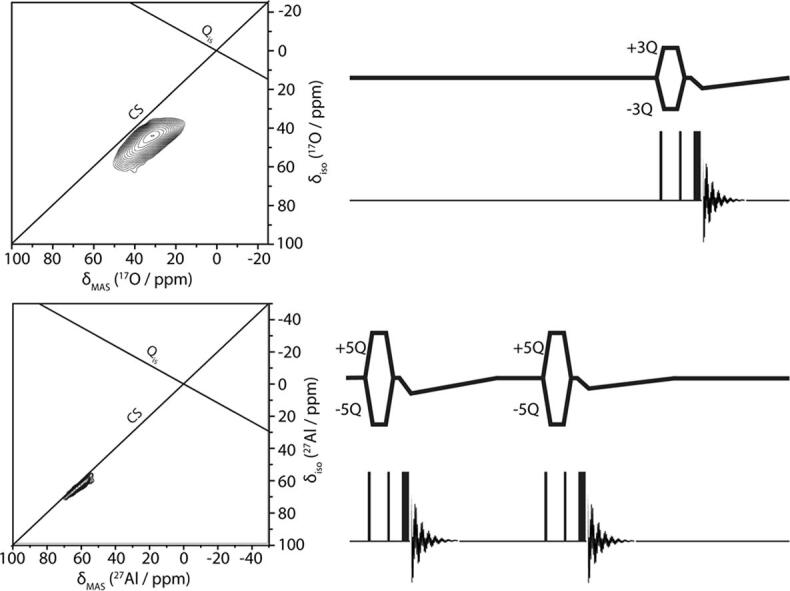

## 1 Introduction

Solid-state NMR is the preferred method for the identification of different chemical environments in non-crystalline samples with atomic resolution, particularly those containing quadrupolar nuclei. In inorganic-materials chemistry, there are a large number of relevant NMR-active nuclei which are commonly used for this purpose. Solid-state NMR is well-suited to characterize a wide variety of material applications including cements [1], [2], geopolymers [3], batteries [4], [5], [6], [7], [8], biomaterials [9], [10], [11], [12], [13], aluminosilicates [14], [15], [16], [17], minerals and other disordered solids [18], [19], [20], [21], [22], [23], [24], [25], [26], [27] where nuclei such as ^11^B, ^17^O, ^27^Al and ^29^Si are important probes into the disorder present. These nuclei span the periodic table, and, accordingly, have a wide variety of quantum spin-numbers, natural abundances, sensitivities, and relaxation rates can vary by ~6 orders of magnitude [28], [29]. There are a relatively small number of multidimensional experiments that are broadly used for the characterization of inorganic materials because the resonances may be very broad, and/or the relaxation time may be quite long. One of the most useful experiments for half-integer quadrupolar nuclei is the Multiple-Quantum Magic Angle Spinning (MQMAS) experiment [30], [31], [32], [33], [34], [35], [36], [37], [38].

**Fig. 1:**
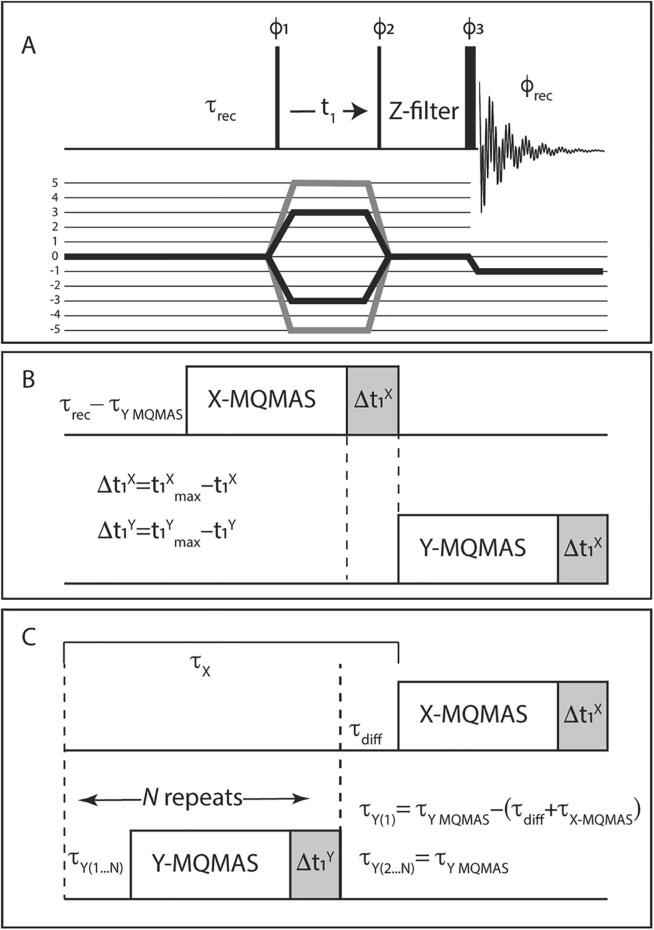
Multiple-receiver MQMAS NMR Pulse Sequences. (A) A schematic for a 3-pulse Z-Filtered MQMAS NMR experiment with a 3Q (black) and 5Q (grey) coherence selection diagram. The 3Q or 5Q half-integer quadrupolar MQ coherence is selected by nested or cogwheel phase cycling. The pulse duration and nutation frequency are controlled independently, where the final central-transition selective pulse is shown with longer duration. (B) Two complete MQMAS experiments, hereafter abstracted as boxes, can be acquired sequentially on isolated frequencies, X and Y, if the recovery delay is made to be the same for both nuclei. τ_Y MQMAS_ is the duration of the MQMAS block for the Y channel, and Δ*t*_1_^X,Y^ is *t*_1_^max^ – *t*_1_ for each respective channel. (C) When the longitudinal relaxation time (*T*_1_) is very different between the nuclei, an integer number of repeats can be acquired on the fast-relaxing nucleus during the recovery delay of the other nucleus. This results in *N* more transients collected on the Y (child) channel than on the X (parent) channel.

The MQMAS experiment is applied for different multi-quantum (triple-quantum (3Q), quintuple-quantum (5Q), septuple-quantum (7Q), and nonuple-quantum (9Q)) to single-quantum (1Q) auto-correlation combinations. There are several variants of the MQMAS experiment [35], where the 3-pulse Z-filtered variant is shown in Fig. 1A [36]. The MQMAS pulse train selects different coherences by allowing only selected pathways with phase cycling, where the 3Q and 5Q pathways are shown in Fig. 1A in black and grey, respectively. MQMAS is usually applied one nucleus at a time, generally with a single or double channel probe, where the X channel is tuned to the nucleus of interest and the ^1^H channel may be used for polarization transfer, such as Cross Polarization (CP), Dipolar Heteronuclear Multiple Quantum Coherence (D-HMQC) or INEPT, to discern or filter for the proximity to ^1^H nuclei [39], [40], [41], [42], as a step in dynamic nuclear polarization (DNP) enhanced experiments [43], [44], or when ^1^H decoupling is needed for high resolution [45]. When multiple nuclei are investigated the probe is re-tuned for each nucleus and observed on a single receiver of the NMR spectrometer.

The rate of data acquisition varies drastically between samples and among detected nuclei due to the variation in longitudinal relaxation times. There are many approaches to speed up the acquisition of NMR experiments such as signal enhancement by dynamic nuclear polarization (DNP)[46], and relaxation enhancement using paramagnetic dopants [47]. There are even set-ups that use multiple samples and MAS modules with a stepper motor to optimize the use of the instrument for slow relaxing nuclei [48]. Experiments with multiple direct acquisitions have been used for small molecules in solution [49], [50], [51] to increase the throughput of multi-dimensional experiments. Spectrometers with multiple receivers have previously been available as specialized equipment where the multiple receivers are used to characterize small molecules with many-at-once experiments [49]. It is now the standard for commercial spectrometers supplied by Bruker to have a receiver for every channel, lowering the barrier for utilizing multiple receivers so such experiments should become more commonplace. However, it is not yet clear how to best exploit this capability efficiently for solids, although some examples are starting to emerge using spin ½ nuclei for MAS NMR of proteins [52], [53], [54] and on a 5-frequency (^1^H, ^19^F, ^31^P, ^27^Al, and ^13^C) probe where 3 receivers are used to measure CP experiments simultaneously using parallel acquisitions [55].

Here, we present the acquisition of interleaved MQMAS experiments that use multiple receivers to improve spectrometer throughput. Interleaving experiments should be broadly applicable to single nucleus experiments such as MQMAS and to other experiments where the recovery delay is not dependent on a shared nucleus.

## 2 Experimental

An ^17^O doped hydrous alkali aluminosilicate (N-A-S-H) gel, 1.18Na_2_O•5SiO_2_•Al_2_O_3_, denoted as “gel B”, where the ^17^O is enriched to 26–31 wt%, was prepared according to Walkley et al. [56] and packed into a standard 3.2 mm Bruker rotor. There are four Al species, two Al^IV^ and two Al^VI^. The Al^IV^ sites have fitted isotropic chemical shift at 61.7 ppm and two C_Q_ values of 1.4 and 1.9 MHz, whereas the Al^VI^ sites have isotropic chemical shifts of 8.9 and 11.1 ppm with C_Q_ of 3.6 and 2.6 MHz, respectively. There are three O species; Si-O^−^-Si, Si-O^−^-Al^IV^, and H_2_O. The two respective silicate sites have a fitted isotropic shift at 39 and 33 ppm with a C_Q_ value of 1.26 and 1.00 MHz, where the water has an isotropic shift at –9.8 ppm and a C_Q_ of 0.55 MHz. A thorough analysis of the spectral features is found in Walkley et al. [56].

A Bruker Avance III HD spectrometer operating at 16.4 T (700.1 MHz ^1^H frequency) equipped with 2 receivers using Topspin 3.5 patch level 6 was used with a 3-channel 3.2 mm Bruker MAS probe in triple channel mode. The spacing of the inductor coils on the HCN trap circuit was increased to move ^13^C (~175 MHz) and ^15^N (~70 MHz) to X = ^27^Al (~183 MHz) and Y = ^17^O (~95 MHz). Many X/Y combinations are possible, so it is useful to consider some representative nuclei that can be probed using MQMAS. These nuclei fall into roughly four frequency ranges with a 16.4 T instrument, where ^7^Li, ^11^B, ^71^Ga, and ^87^Rb, make a high (272–223 MHz) frequency group; ^23^Na, ^27^Al, ^45^Sc, ^59^Co, ^69^Ga and ^93^Nb make a medium–high (185–165 MHz) group; ^17^O and ^35^Cl (95–68 MHz) is medium–low; and ^25^Mg, ^39^K, ^43^Ca, ^95^Mo, and ^97^Mo, (48–32 MHz) make up the final, low frequency group. The “HCN” trap circuit can be tuned to the nuclei in the medium–high group on the X channel and the medium-low and the upper range of the low frequency group (^43^Ca and ^97^Mo) on the Y channel according to the vendor specifications and confirmed with a network analyzer. Similarly, an “HPC” trap can be used to tune the high nuclei on the X channel, with the medium-high nuclei tuned on the Y channel. These two traps theoretically enable a wide range of possible nuclei combinations, if the power handling is adequate in such a configuration. All experiments were performed at ambient temperature and 20 kHz spinning frequency. The nutation frequencies were individually optimized for double and triple resonance mode on standard samples (Yttrium Aluminum Garnet (YAG), and 10% ^17^O doped water) and then confirmed on the sample. In triple-resonance configuration (HXY), the excitation, conversion and central-transition selective pulses were 6.3, 2.3, and 11 µs for the X (^27^Al) channel where the maximum solution nutation frequency, *ν*_1_^max^_,_ is ~56 kHz, and 7.0, 2.4, and 11 µs for the Y (^17^O) channel (*ν*_1_^max^ ~ 67 kHz). For the double resonance configuration (HX) excitation, conversion and central-transition selective pulse were 5.2, 2.0, and 11 µs for ^27^Al (*ν*_1_^max^ ~ 75 kHz), and 6.2, 2.0, and 11 µs for ^17^O (*ν*_1_^max^ ~ 71 kHz). In both triple-resonance (HXY) and double-resonance (HX) configurations for ^17^O and ^27^Al the 11 µs central-transition pulse corresponded to a solution nutation frequency of 7.57 kHz ensuring the central transitions of all sites were excited. The experiments utilize the parent–child architecture to use multiple receivers provided by the vendor. Briefly, two (or more) files are created with independent direct-dimension acquisition parameters, *i.e.* receiver routing, frequency, acquisition time, sweep width, and the number of points in the FID. One file, the parent, is used to control the children and all other parameters, such as the nutation frequencies, delays and indirect dimension acquisition parameters are stored in the parent file. The 3QMAS + 3QMAS parent (child) experiments were collected with 144 *t*_1_ FIDs each with 144 (288) co-added transients with a *t_1_* increment of 25 (12.5) µs using the States method [57] for sign discrimination in the indirect dimension and a recovery delay of 1.0 (0.4) s, for a total of 5.9 h of experiment time. The 3QMAS + 5QMAS experiments were collected with 102 *t*_1_ FIDs each with 1440 (2880) co-added transients with a *t*_1_ increment of 25 (12.5) µs and a recovery delay of 1.0 (0.4) s, for a total of 42.0 h of experiment time.

The data was processed with 200 Hz of Lorentzian line-broadening in both dimensions, using the shear transform provided with the Topspin software for the indirect dimension [36]. The child experiment will likely have the wrong nucleus and timing listed for the indirect dimension. A constant (cnst1) is used in the parent experiment to control the timings for the indirect dimension of the child experiment. Topspin assumes that the indirect dimension timings are the same for the parent and child experiment, so the command “s swh” is used to correct the spectral width in the child experiment. The shear transform will not be performed correctly unless the child experiment has the correct acquisition parameters. There is no need to split the experiments, or any other preprocessing beyond correcting the time step, since the data is collected into separate files.

## 3 Pulse Sequence Design

MQMAS experiments are typically single-channel autocorrelation experiments (Fig. 1A) where a coherence pathway is chosen by phase cycling the three pulses based on the nuclear spin and desired multiple-quantum coherence. If the nuclei are separated in their NMR frequency by several MHz, applying RF pulses at the far-away frequency will not directly affect any other nuclei. Therefore, two MQMAS experiments running in succession are not expected to interfere with one another as long as the probe remains tuned, and the frequencies are sufficiently far from one another. In any case, the experiments for the separate nuclei are not run simultaneously to avoid any possible unintended recoupling for this implementation of multiple receiver experiment.

The simplest interleaved experiment (Fig. 1B) is better described as a staggered experiment. The signal from one of the nuclei (Y, in this example) is collected immediately after the other (X) with the result that both nuclei have the same recovery delay. It is likely, however, that one nucleus relaxes faster than the other. In fact, the fast-relaxing nucleus may relax several orders of magnitude faster than the slow-relaxing nucleus. It is thus possible to acquire an integer number, *N*, experiments on the fast-relaxing nucleus while waiting for the slow-relaxing nucleus (Fig. 1C). The slow-relaxing nucleus is assigned to be the “parent” dataset, and the faster nucleus is then designated as the “child” dataset (using the definition for parallel acquisitions as provided by the vendor).

Care must be taken to prevent large variations in the recovery delay of both experiments, and thus artificially introduce t_1_ noise into either experiment. Because the experiments are run as a Parent-Child process, the timings for one are intertwined in the other. Usually, the recovery delay is started immediately after the acquisition time, which ensures that the time between the last pulse of one transient and the first pulse of the next is the same. As the evolution time (*t*_1_) grows, the time to complete the acquisition also grows with it. In these interleaved experiments the recovery delay (τ_x_) of the parent experiment is composed of “*N*” child experiments. If the child experiment grows longer for every completed *t*_1_ FID, then the parent recovery delay τ_x_ will also increase by *N*•*t*_1_. For a reasonably large “*N*” and/or *t*_1_^max^, this contribution may become significant. This time difference can be accounted for by waiting a time *N•*Δ*t* where Δ*t* = *t*_1_^max^-*t*_1_. The timing between child experiments could also vary, for example, if τ_diff_ + τ_X-MQMAS_ is on the order of τ_Y_, then the actual recovery delay will be ~2•τ_Y_ every *N*^th^ transient. This problem in the child experiment’s timings would also occur if the compensation time for the parent (*N*•Δ*t*) were to be added on to τ_diff_. To minimize the chance that the recovery delay is significantly altered, each FID acquisition is made to last a constant time by adding the delay Δ*t* after the transient is collected. Additionally, the first delay after parent acquisition is either reduced by τ_diff_ and the time needed for the parent experiment (τ_Y_^(1)^ = τ_Y-MQMAS_–(τ_diff_ + τ_X-MQMAS_)), or is set to 0. It is prudent to alter the child experiment recovery delay to fit well into the parent’s recovery delay since the experiment time is only dependent on the parent timings (*i.e.* τ_diff_ is minimized). Until each channel can be run independently, reducing the possibility for any variation in the recover delays is the best way to limit the introduction of *t*_1_ noise. If the parent experiment requires acquisition times longer than the recovery delay of the child, and *N* is large, dummy scans on the child experiment might mitigate the *t*_1_ noise, but we did not explore that possibility.

Several experiments can be built onto the same framework, where only the phase cycle is changed to select the different coherence pathways. Here we will explore options for observing two spin 5/2 nuclei simultaneously where the options for parent + child MQMAS experiments are 3Q + 3Q, 5Q + 3Q, 3Q + 5Q, and 5Q + 5Q (where the notation refers only to the MQ portion of the MQ-SQ experiment). Therefore, options are defined in the pulse program to quickly switch between the phase cycles of these experiments (https://wrap.warwick.ac.uk/138627). The phase cycle for other spin numbers, MQ excitation, or coherence pathways can easily be appended or substituted.

The multiple-quantum coherence is selected solely through phase cycling. The phase cycle, ideally, provides a full coherence pathway selection in as few acquisitions as possible. This lets the investigator more efficiently utilize the instrument time across samples that are “sampling limited” and those that are “sensitivity limited”. Sampling limited experiments are defined as those that require more repetitions than necessary as dictated by the sensitivity just to finish the phase cycle, and sensitivity limited experiments require more than one repetition of the phase cycle to reach the needed sensitivity [58]. The phase cycle of the MQMAS experiment changes depending on the desired multi-quantum coherence. In this work, we have only considered 3Q and 5Q coherences of sensitivity limited samples, see Appendix for phase cycles; a comprehensive review of the phase cycling of such experiments can be found in Millot, Hajjar and Man [59]. The least number of co-added transients determined to date using cogwheel phase cycling to robustly select the desired pathway is 36 for a 3Q MQMAS (60 for a 5Q MQMAS) experiment [59], [60], [61]. When collecting nested MQ experiments, care should be taken so that the phase cycle for both experiments is completed at the same time. We must find a common product, where both experiments complete an integer number of phase cycles. For example, we have

(1) $$k*{\mathrm{ns}}_{3Q}=l*{\mathrm{ns}}_{5Q}$$

Once the cogwheel phase cycling number is substituted for the number of steps, we have:

(2) k∗36=l∗60

The smallest common integer product is when *k* = 5 and *l* = 3, so the total number of steps must be a multiple of 180 to ensure that both experiments complete a full phase cycle. However, in the case where multiple repetitions are possible, as shown in Fig. 1C, there is an extra consideration. In our sample, the longitudinal relaxation for the ^27^Al (*T_1_* ≅ 0.35 s) (*T*_recovery_ = 0.50 s) is a little more than twice as fast as for ^17^O (*T_1_* ≅ 0.8 s) (*T*_recovery_ = 1.05 s). Here, *T*_recovery_ is ~1.3*T_1_ which gives the highest sensitivity in the shortest time assuming full saturation [62]. Therefore, the child (fast, ^27^Al) experiment steps through its phase cycle *N = 2* times faster than the parent. The child’s minimum phase cycle is effectively 30 (60/2) from the perspective of the parent experiment, where the parameters are controlled. So, “*k*” is the important factor because it determines the steps of the parent experiment, and the steps in the child will be k*ns_3Q_*N. In the particular case of two transients of a 5QMAS experiment ^27^Al interleaved into a 3QMAS experiment on ^17^O demonstrated in this work, the multiplier for the number of steps found by finding “*k*” in the following equation.

(3) k∗36=l∗60/N=l∗30

The combined experiment will still require a multiple of 180 steps in the slow (parent) experiment, unless *N* is a multiple of 5 which reduces the minimum parent steps down to 60. In our example, 360 (6 * 30 * 2) transients are collected in the fast (child) experiment for every 180 collected in the parent. To most efficiently fit the experiments together it may be advantageous to have several options for the number of steps for the cogwheel phase cycle of each channel. A topspin macro written in python is available at (https://wrap.warwick.ac.uk/138627) to quickly determine the minimum number of steps needed by the parent given the length of the two separate phase cycles and child repetitions (*N*).

## 4 Results and Discussion

**Fig. 2:**
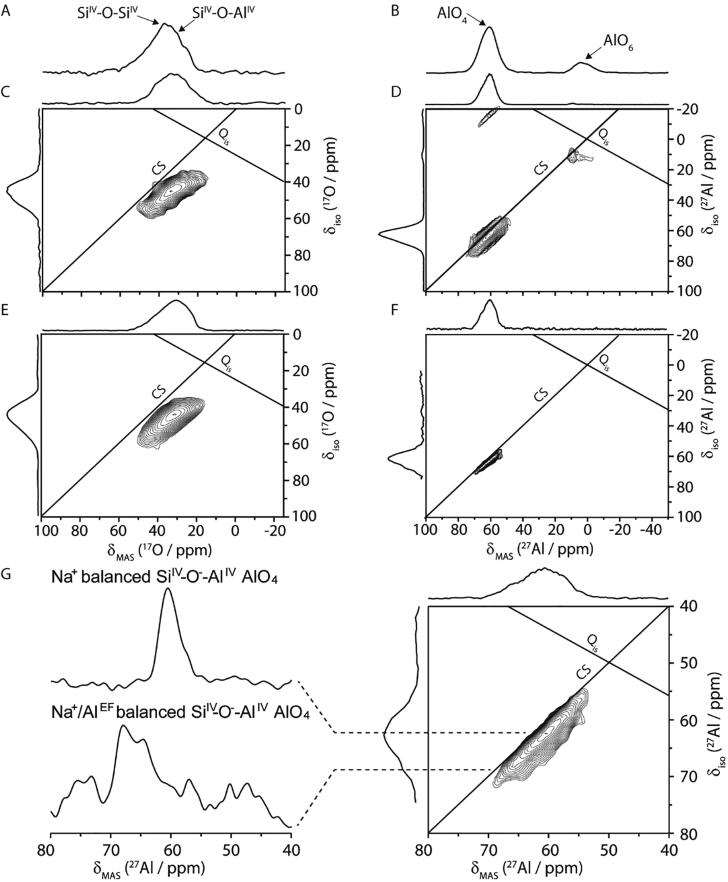
^17^O (A) and ^27^Al (B) Double-Receiver Hahn-echo MAS NMR spectra of a NASH glass gel 1.18Na_2_O•5SiO_2_•Al_2_O_3_. The double-receiver experiments, as shown in Fig. 1C, for 3QMAS on ^17^O (C) and the 3QMAS on ^27^Al (D) are acquired simultaneously, likewise, 3QMAS on ^17^O (E) and 5QMAS on ^27^Al (F) are also acquired simultaneously. (G) A focused view of the AlO_4_ sites of the ^27^Al 5QMAS experiment. Data were collected on a spectrometer operating at 700.1 MHz ^1^H frequency and a spinning rate of 20 kHz processed with “normalized” shearing which produces a diagonal for the chemical shift (CS axis) and the Quadrupolar interaction (Q_IS_ axis). The vertical skyline projections correspond to the sheared MQ coherence, and the horizontal skyline projection is the 1Q, directly observed spectrum. The 3Q3Q experiment (C,D) required 5.9 h, while the 5Q3Q experiments (E,F) required 42.0 h. Positive contours are with the first contour drawn at 6 times the standard deviation of the noise.

An ^17^O-doped alumina-silicate NASH glass gel 1.18Na_2_O•5SiO_2_•Al_2_O_3_[56] was found to provide good signal in a reasonable amount of time for both nuclei as seen by the Hahn echo 1D MAS NMR experiments (Fig. 2A and 2B). The 3Q + 3Q experiment produces two 3QMAS experiments, one for ^17^O and one for ^27^Al (Fig. 2C and D). In this sample, ^17^O is the low frequency, and must be the Y-channel on the NMR probe. The ^27^Al relaxes about twice as fast as ^17^O in this sample, so ^27^Al is designated as the “child” experiment.

For this sample, the ^27^Al 3QMAS experiment has a higher S/N ratio than necessary because the ^17^O experiment needed extra averaging for adequate sensitivity. However, this still resulted in insufficient resolution to clearly distinguish the distinct Chemical Shift (CS) and Quadrupolar induced shift (Q_is_) tailing in the ^17^O MQMAS to unambiguously identify the presence of the Si^IV^–O–Al^IV^ and Si^IV^–O–Si^IV^ sites as seen before [56]. The sensitivity for the ^27^Al 5QMAS experiment more closely matches the sensitivity required for the ^17^O 3QMAS experiment. The larger number of scans needed for sensitivity in the 5QMAS experiment also increases the signal to noise in the ^17^O 3QMAS spectrum. The better ^17^O sensitivity allows for the identification of distributions of both oxygen sites observed in Walkley et al. [56]. In the 5Q + 3Q experiment, the AlO_6_ site (~10 ppm) has poor sensitivity due to the reduced MQ excitation efficiency, typically 5 or 6 times less efficient [32], [41], but it is still identifiable at low baseline display levels. However, there is a marked improvement in the apparent resolution, *e.g.* the spacing between peaks in the indirect dimension, of the AlO_4_ site (~65 ppm). While the primary AlO_4_ resonance representing the Na^+^ balanced Si^IV^–O^−^–Al^IV^ site is clear in both ^27^Al 3Q- and 5QMAS spectra, the secondary AlO_4_ site is not clearly resolved in the 3QMAS data presented here. The enhanced resolution afforded by the ^27^Al 5QMAS has made it possible to resolve the Na^+^/Al^EF^(extra framework Al) balanced Si^IV^–O^−^–Al^IV^ AlO_4_ resonance without resorting to biaxial Q-shearing of the MQMAS data [63]. As shown in Fig. 2G, a shoulder can be observed in the isotropic dimension of the AlO_4_ site. The increased contributions from CS broadening and the decreased quadrupolar coupling from being at a higher field than as for the results presented in Ref. [56] at 14.1 T is likely the explanation as to why both sites are unresolved in the ^27^Al 3QMAS spectrum in Fig. 2. Narrowing at higher quantum coherence is expected for samples where the multiple-quantum frequency scaling is greater than the inhomogeneous contribution to the linewidth [41], and may resolve additional sites in those cases. Since 5QMAS experiments are less sensitive to homogeneous broadening than 3QMAS experiments [64], a 5QMAS experiment may be preferred in systems where *I* ≥ 5/2 and there are strong homogeneous interactions. In general, the sensitivity and evolution times needed will be different for the various nuclei. Currently, the experiments must have the same number of *t*_1_ FIDS (*i.e.* rows), but the timestep between rows is independent. So, sensitivity can be traded for resolution to some extent on one channel, while no compromise is needed for the other.

The multi-MQMAS experiments were calibrated using the equivalent single channel experiments, but with the hardware set up for the triple resonance experiments. This provides an estimate of the sensitivity, and which variants of the experiment could be collected. The recovery delays for each nucleus were adjusted so that a whole number of child experiments fits within the parent recovery delay. Effectively, the value of the parameter τ_diff_ (which varies according to the recovery delay of the child experiment) shown in Fig. 1C is minimized by changing the recovery delay of the child experiment.

**Fig. 3:**
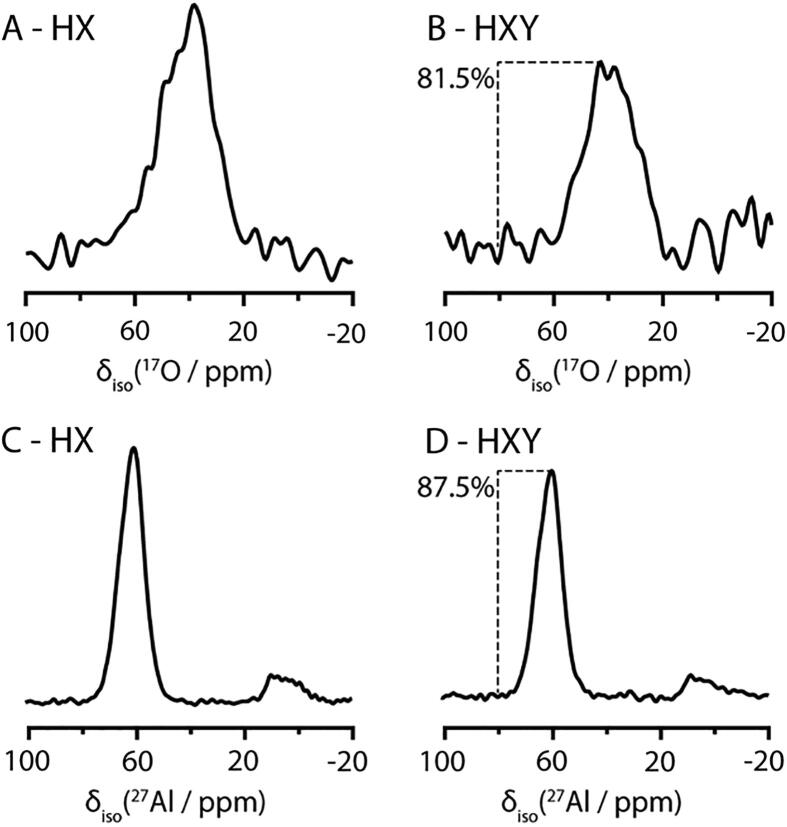
3Q MQMAS filtered (*t*_1_ = 0) NMR efficiency comparison for ^17^O (A,B) and ^27^Al (C,D) in a NASH glass gel 1.18Na_2_O•5SiO_2_•Al_2_O_3_ glass between double resonance (HX, A and C) and triple resonance (HXY, B and D) on a spectrometer operating at 700.1 MHz ^1^H frequency, with spinning rate of 20 kHz, where 2048 transients were collected for ^17^O (A,B) and 4096 transients were collected for ^27^Al (C,D). The signal intensity of the triple resonance experiments compared to the identical experiment by overlay is indicated in B and D.

It is difficult to quantify the amount of time saved by simultaneously acquiring MQMAS experiments in triple resonance due to the many complicating factors. The key factor in the success of an MQMAS experiment is the achievable nutation frequency of the reconversion pulse [35], [36], which may limit the number of sites excited into MQ coherences and will be affected by the complexity of the probe circuit. Additionally, the NMR probe will lose sensitivity in triple resonance mode, especially for the low-frequency Y-channel, as compared to double or single channel tuning. The power handling between the two probe tunings modes will affect the optimization of the nutation frequency. We have attempted to quantify the loss between triple mode (HXY) and double mode (HX) for these MQMAS experiments by tuning the probe to ^1^H-^27^Al, ^1^H-^17^O, and ^1^H-^27^Al-^17^O, and optimizing each experiment in turn. The first row (*t*_1_ = 0) of an MQMAS experiment with 2048 transients for ^17^O and 4096 transients for ^27^Al was then collected for each configuration. As seen in Fig. 3, there is a loss of 15–20% (18.5% by overlay) for the ^17^O (low-frequency, Y-tuned), and a 10–15% (12.5% by overlay) loss for the ^27^Al (middle frequency, X) channel as compared to the probe in double resonance. The nutation frequencies used for MQ coherence excitation are higher than typical spin-½ triple resonance CP experiments, for example in biological applications. However, the pulse durations are also much shorter in comparison. Additionally, spectra collected individually in triple-resonance mode are indistinguishable from those collected by interleaving experiments. The extra power deposited in the probe by the third channel did not cause deterioration in spectral quality, which would be indicated by arcing or an extra, unexpected loss of signal intensity. The duty cycle for these particular experiments is quite low, so caution should be practiced for samples with faster recovery times.

The time required to reach the same sensitivity between the two probe tuning modes can be calculated according to the following equation:

(4) $$t_{{\mathrm{Expt}}_{1}}={\left(I_{0}/I_{1}\right)}^{2}t_{{\mathrm{Expt}}_{0}}$$

where *I*_0_/*I*_1_ is the ratio of the intensities of the reference and trial experiments. In the worst case of a 20% loss for all experiments (*I*_1_/*I*_0_ = 0.8), the time required to get back to the same signal to noise requires 1.56 [(1./0.8)^2^] times longer than the standard setup, where the best case of 10% loss requires 1.23 times longer. Using the signal intensities for the worst-case nested experiments relative to the two conventionally acquired experiments, the multiple receiver experiment takes ~78% (1.56/2) as long to collect as the two separate, equal quality experiments. If we assume for convenience that the two nuclei have equal efficiency losses between the two modes of acquisition, the two modes will break even when *t*_Expt 1_/*t*_Expt 0_ = 2. This implies that the efficiency for the triple resonance mode experiments should be greater than *I*_1_*/I*_0_ = ($1/\sqrt{2}$) ≅ 70% compared to the double resonance experiments for efficient data collection.

The receptivity (and/or % labelling) also contributes directly to the time-spent, or feasibility of, collecting any MQMAS experiment. With that said, many experiments are time-prohibitive on their own but may be a reasonable use of instrument time when paired with a second time-prohibitive experiment. In the future, it may be possible to string together several different experiments on the high-sensitivity nucleus while collecting other MQMAS experiments on the less sensitive nucleus during the recovery delay. For example, the number of acquired transients and rows in the indirect dimension need not be tied as closely to one another as they are here and could perhaps be made independent. In an ideal scenario, one experiment could be set to average in the background, as if it were a separate spectrometer, while other experiments are optimized and/or acquired.

## 5 Conclusions

We demonstrate a way to more efficiently utilize NMR instrument time to measure MQMAS experiments for half-integer quadrupolar nuclei with multiple receivers and multiply tuned probes. The experiments presented here are a demonstration of the power of multiple receivers in solid-state NMR of materials [1], [2], [3], [4], [5], [6], [7], [8], [9], [10], [11], [12], [13], [14], [15], [16], [17], [18], [19], [20], [21], [22], [23], [24], [25], [26], [27] with applications to biological samples also possible [12], [13], [65], [66]. Embedded looping was previously presented for solution and solid-state NMR multiple receiver experiments and is a powerful tool to collect full NMR datasets for small molecules in a short time [49], [50], [51], [52], [53], [54]. The core concept of these experiments should be adaptable to many different experiments in materials solid-state NMR such as mixed HETCOR + MQMAS, mixed dimension experiments, (2D + 1D), and more sophisticated MQMAS experiments.

## CRediT authorship contribution statement

**Samuel J. Page:** Conceptualization, Methodology, Software, Writing - original draft, Visualization. **Angelo Gallo:** Conceptualization, Methodology, Software, Writing - original draft, Visualization. **Steven P. Brown:** Supervision, Resources, Funding acquisition, Writing - review & editing. **Józef R. Lewandowski:** Supervision, Resources, Funding acquisition, Writing - review & editing. **John V. Hanna:** Supervision, Resources, Funding acquisition, Writing - review & editing. **W. Trent Franks:** Conceptualization, Methodology, Software, Writing - original draft, Visualization, Supervision, Project administration.

## Declaration of Competing Interest

The authors declare that they have no known competing financial interests or personal relationships that could have appeared to influence the work reported in this paper.
