# Supplementary material for: Simultaneous MQMAS NMR Experiments for Two Half-Integer Quadrupolar Nuclei
Source: J Magn Reson. 2020 Nov;320:106831. doi: 10.1016/j.jmr.2020.106831 (PMC7661836; doi:10.1016/j.jmr.2020.106831)
Supplement: Supplementary data 1 [file mmc1.docx]

**Appendix/Supplementary Information**

The nested cogwheel phase cycle for the MQMAS experiments used are as follows [59]:

Phases are numbered sequentially, and “φ_2_= {0}*6” means that φ_2_ is 0 for six repetitions.

3Q MAS (I=5/2; ns_3Q_=36)

φ_1_= 0, 60, 120, 180, 240, 300

φ_2_= {0}*6, {60}*6, {120}*6, {180}*6, {240}*6, {300}*6

φ_3_= 0

φ_rec_= +3*φ_1_ -3*φ_2_ + φ_3_

5Q MAS (I=5/2; ns_5Q_=60)

φ_1_= 0, 36, 72, 108, 144, 216, 252, 288, 324

φ_2_= 0

φ_3_= {0}*10, {60}*10, {120}*10, {180}*10, {240}*10, {300}*10

φ_rec_= +5*φ_1_ - 5*φ_2_ + φ_3_

The Bruker pulse code, datasets, processing parameters, and Topspin python extension presented in this paper are available online at ([https://wrap.warwick.ac.uk/138627](https://wrap.warwick.ac.uk/138627?fbclid=IwAR2tR4brqYBLnG5oYBS-FLkPj3U_Aqovxm-6r5JdcGgb-c46b3jqMp4eGEs))
